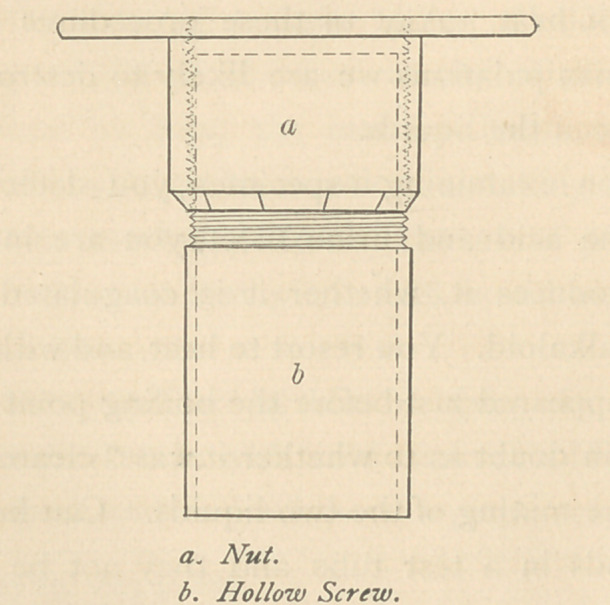# A Simple Microtome

**Published:** 1885-01

**Authors:** C. C. Webster

**Affiliations:** Chicago


### ARTICLE IV.

A SIMPLE MICROTOME. *By* C. C. WEBSTER, M. D.

Several years ago, Mr. O. B. Marsh, of Binghampton, N. Y., made, at my suggestion, the herein described instrument. I have found it very useful, particularly in cutting sections of malignant growths for diagnosis. The frequent necessity for such an instrument leads me to publish the description, with

*a. Nut.*

*b. Hollow Screw.*

---

*London Lancet, April, 1883.

the hope that the reader, if he choose to make a duplicate, will find it as serviceable to him as the original has been to me. The proportions, given in the cut, are nearly the same as those of my instrument, but exact measurements are unimportant, as several professional acquaintances have made different sizes without materially affecting its usefulness.

There are two parts to the microtome. It is a hollow screw, in which the object is to be embedded. It is either to be packed in with cork, liver or some suitable material, imbedded in soap, paraffine, etc., or it may be mounted on cork with mucilage and then hardened. When in the cut, the cork can be slipped into the tube. However the object may be arranged, the portion to be cut must project above the upper end of the tube.

The nut *a* is provided with a broad flat top, which serves as the surface for guiding the razor. This top may be faced with plate glass, and for convenience its edge may be milled. The nut and screw are cut with a thread fifty to the inch. The accuracy of this thread determines the usefulness of the instrument. It should be cut in a lathe, and be as nearly perfect as possible.

In using the instrument, one or two preliminary cuts should be made to level off the top of the object, each time turning down the nut, thus causing the object to project. After the top of the object is leveled, the desired thickness for the section can be measured off by a vertical mark cut on the screw and spaces marked on the lower edge of the nut. If these spaces are tenths of the circumference, turning the nut one space will give section of one-five-hundredths of an inch. Such a section is too thick for such purposes; therefore, it will be found best to mark the nut with more spaces, or turn it some fraction of a space.

Chicago.

## Figures and Tables

**Figure f1:**